# Impact of Frailty on the Duration and Type of Speech-Language-Hearing Therapy for Patients With COVID-19

**DOI:** 10.7759/cureus.81976

**Published:** 2025-04-09

**Authors:** Shinichi Watanabe, Takaaki Sakurai, Takahiro Kanaya, Takumi Iwasaki, Hyosuke Oshima, Tetsuya Furukawa, Tomohiro Yoshikawa, Seichi Nakahashi

**Affiliations:** 1 Department of Rehabilitation, Gifu University of Health Science, Gifu, JPN; 2 Department of Rehabilitation, National Hospital Organization, Nagoya Medical Center, Nagoya, JPN; 3 Health Promotion, Shibetsu Town Health and Welfare Center, Hokkaido, JPN; 4 Department of Rehabilitation, National Hospital Organization, Hokkaido Medical Center, Hokkaido, JPN

**Keywords:** covid-19, frailty, oral intake, speech-language-hearing therapy, standard rehabilitation

## Abstract

Introduction

This study aimed to explore the standard rehabilitation for patients with COVID-19 who did not receive invasive ventilation treatment and to descriptively clarify the timing and content of speech-language-hearing therapy (SLT), categorizing patients based on the presence or absence of frailty before symptom onset.

Methods

This retrospective observational study included patients aged ≥18 years admitted for COVID-19. SLT was performed at each hospital as per a common protocol. The exposure variable was frailty versus nonfrailty (defined as a clinical frailty scale score of ≥4). Multiple linear analyses adjusted for baseline characteristics were used to determine the link between frailty and mean SLT time. We investigated mean SLT time (min/day), total SLT time, total SLT days, and SLT content per session from weeks one to four.

Results

Of the 254 patients, 207 and 47 were assigned to frail and nonfrail groups, respectively. The mean SLT time in the frail group was significantly higher than that in the nonfrail group. Furthermore, total SLT time, total SLT days, functional oral intake scale (FOIS) score at hospital discharge, incidence of hospital-acquired pneumonia, and discharge to home were significantly longer and higher in the frail group than those in the nonfrail group. The frail group performed considerably more indirect and direct swallowing exercises than the nonfrail group.

Conclusions

Daily SLT time and total SLT days on which SLT was performed were substantially longer in the frail group, with higher rates of direct and indirect swallowing exercises.

## Introduction

COVID-19, a viral pandemic caused by the severe acute respiratory syndrome coronavirus 2, originated in East Asia and has quickly spread globally [1]. In patients with severe COVID-19, prolonged tracheal intubation and mechanical ventilation have led to dysphagia development [2]. Although dysphagia is common in patients who undergo long-term endotracheal intubation in the intensive care unit [3], it remains unclear whether it is due to intubation, viral infection, or both. At present, no established approach exists for treating dysphagia in patients with severe COVID-19 [4].

The COVID-19 pandemic has highlighted the importance of dysphagia rehabilitation [5], with speech-language-hearing therapy (SLT) playing a key role in managing patients with dysphagia [6]. Dysphagia symptoms can be assessed through clinical tests, such as cough tests. However, SLTs face an increased risk of exposure to COVID-19 infection due to saliva, respiratory droplets, and aerosols generated during dysphagia rehabilitation, which raises concerns about infection and physical strain [7].

While some studies in Japan have examined the impact of feeding and swallowing disorder assessment and exercise in patients with COVID-19 [8], detailed investigations on the workload of feeding and swallowing rehabilitation, such as duration, frequency assessment methods, and exercises based on patient severity are still lacking.

Early rehabilitation for dysphagia in more severe COVID-19 patients is anticipated to involve a multidisciplinary team providing bedside care, requiring significant time and manpower [9]. However, the duration actually spent on SLT rehabilitation interventions for patients with COVID-19 has not been reported, and the interventions that are being conducted are unclear [8]. Moreover, with the rising and aging population of patients with dysphagia, especially those who are frail, the specifics of rehabilitation provided to those with frailty and COVID-19 must be clarified.

This study aimed to investigate the standard rehabilitation for patients with COVID-19 who are not on invasive ventilation, specifically examining the timing and content of speech rehabilitation based on the presence or absence of frailty before symptom onset. Focusing on frail patients with COVID-19, this study compared the intensity of feeding and swallowing rehabilitation with that of nonfrail patients, seeking to identify solutions, such as the number of rehabilitation staff assigned to these patients.

## Materials and methods

Study design and patient selection

This multicenter, retrospective study was conducted in two hospitals in Japan. Furthermore, the study was conducted in accordance with the STROBE guidelines [10]. Patients with COVID-19 aged ≥18 years between May 2022 and December 2023 were enrolled. Patients receiving invasive mechanical ventilation, those with contraindications to oral intake owing to tracheostomy, those with gastrointestinal perforation, and those with a lack of communication ability or terminal conditions were excluded.

Data collection

Patient characteristics were surveyed, including age, sex, body mass index, Charlson comorbidity index, COVID severity, days of isolation, pre-hospitalization clinical frailty scale [11], and pre-hospitalization functional oral intake scale (FOIS) [12].

The clinical frailty scale is a clinical judgment-based frailty tool developed for the Canadian Study of Health and Aging [11]. This scale evaluates specific domains including comorbidity, function, and cognition to generate a frailty score ranging from 1 (very fit) to 9 (terminally ill). Herein, a person was deemed frail if the clinical frailty scale score was >4. FOIS, a validated and reliable scale for measuring dysphagia, was examined during SLT rehabilitation. Scores ranged from 1 (nothing by mouth) to 7 (total oral diet with no restrictions), with higher scores indicating improved swallowing function [12].

Primary outcome

The primary outcome was the time spent performing SLT per session (min/session), and the follow-up period was until discharge. As performed in previous studies, the person in charge of performing SLT measured the time spent on the SLT from the start to the end of the SLT (including preparation and rest times) in minutes.

Secondary outcome

Secondary outcome items were total SLT time (min), total SLT days, time to first SLT day, FOIS improvement, FOIS at hospital discharge, hospital-acquired pneumonia, length of hospital stay, discharge to home, and in-hospital mortality. Hospital-acquired pneumonia was defined as pneumonia that developed >48 h after hospitalization and had no initial symptoms at the time of admission [13].

SLT rehabilitation

**Figure 1:**
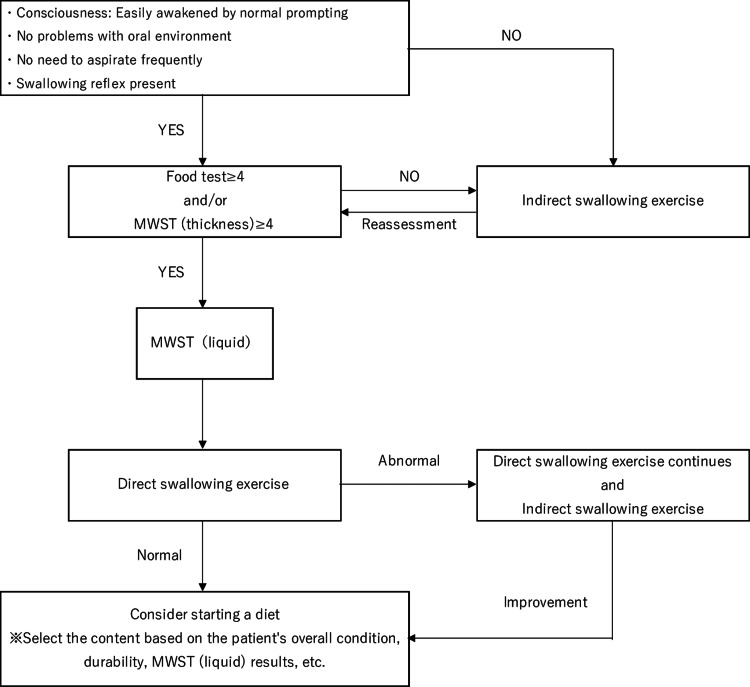
SLT protocol MWST, modified water swallowing test; SLT, speech-language-hearing therapy

A dedicated SLT performed a swallowing assessment under the supervision of a physician in accordance with the Swallowing Clinical Practice Guidelines 2018 [14] and the Expert Consensus on Early Rehabilitation [9]. The patient's level of consciousness, overall condition, swallowing reflex, respiratory condition after swallowing, oral environment, ability to cough or choke, and tongue and laryngeal movements were evaluated based on previous studies as a criterion for oral intake, and a food test [15] and modified water swallowing test [16] were performed according to the flowchart. The attending physician decided whether the patient could start eating based on the evaluation results (Figure 1).

Once the patient was deemed ready to begin eating, the SLT and nurse collaborated to ensure there were no signs of choking, persistent fever, increased airway secretions, decreased level of consciousness, or respiratory decline. The diet was gradually modified from jelly to blended, chopped, and soft vegetable diets until the patient could consume regular food.

If swallowing disorders were detected, the patient was referred to the attending physician, and individualized dysphagia rehabilitation by the SLT began. The intervention continued until discharge or until the patient was able to eat independently. When direct swallowing exercises were deemed challenging based on the initial assessment, the exercises included both active and passive movements of the swallowing muscles, while maintaining the oral cavity, expectorating, and performing ice massage of the throat. A dedicated registered dietitian assessed the patient’s nutritional status and provided recommendations on nutritional methods and amounts based on the patient's swallowing function. If direct training and meal initiation were considered, swallowing endoscopy or radiography was conducted after isolation to evaluate functional disorders and establish goals for oral intake. Table 1 lists the definitions and details of the direct and indirect swallowing exercises [17-19].

**Table 1:** Definition and classification of speech-language therapy

	Definition	Classification
Indirect swallowing training	Indirect therapies do not directly target swallowing retraining but are aimed at treating the underlying disease through pharmacological, surgery or involve muscle strengthening, sensory stimulation, or cortical stimulation by transcranial magnetic stimulation or transcranial direct current stimulation: Teresa et al. 2013 [17].	Oral and pharyngeal care, active and passive movements of organs related to swallowing, throat ice massage, cough training, blowing exercise.
Direct swallowing training	Compensatory strategies while swallowing such as chin tuck or head turn to increase airway protection, augmentative swallowing maneuvers such as Mendelsohn or supraglottic swallowing, or diet modification to reduce the risk of aspiration such as thickened liquids: Teresa et al. 2013 [17].	Adjustment of food type and amount, adjustment of eating pace, limiting bite size, posture adjustment, use of compensatory swallowing techniques.
Cognitive training	Individual training. Guided practice of a set of structured usually standardized tasks, designed to train individuals on relatively well‐defined cognitive processes and abilities such as speed of information processing, attention, memory, or problem‐solving: Bahar-Fuchs et al. 2019 [18].	Paper and pen-based cognitive training: calculation task, verbal fluency task, reciting task, attention process training.
Articulation training	Behavioral treatment of articulation can involve either a direct or an indirect approach. Direct methods focus on the segmental level of speech and aim to alter the identity of phonemes. Indirect methods focus on the suprasegmental level meaning that the strategies are superimposed on individual phonemes or sequences of phonemes and the effect on articulation: Vergara et al. [19].	Oral diadochokinetic, vocalization training, articulation training.

Sub-analysis

We investigated the mean SLT time (min/day), total SLT time, total SLT days, and SLT content (indirect swallowing exercises, direct swallowing exercises, cognitive exercises, and speech and articulation exercises) per session from weeks one to four.

Ethical approval

This research was approved by the ethics committee of each participating hospital and the ethics committee of Nagoya Medical Center (institutional review board approval number: 2022061, date of approval: 9 May 2023).　

Statistical analysis

The baseline data and course factors were compared between the frail and nonfrail groups. Frailty was defined as a Clinical Frailty Scale score of ≥4 before onset. Data are presented as medians with interquartile ranges or numbers with percentages. The Mann-Whitney U test and the χ2 test or Fisher’s exact test were used to analyze continuous and nominal variables, respectively, as appropriate. Multiple linear analyses were performed with age, sex, and Charlson comorbidity index as covariates to evaluate the impact of frailty on the mean SLT time. We analyzed the secondary and other outcomes by performing multiple linear and logistic regression analyses for log-transformed continuous and categorical variables, respectively, using the same covariates used to analyze the primary outcome.

All analyses were performed using JMP (version 13.0; SAS Institute, Cary, NC, USA). Statistical tests were two-tailed, and statistical significance was set at p < 0.05.

## Results

Baseline characteristics

**Figure 2:**
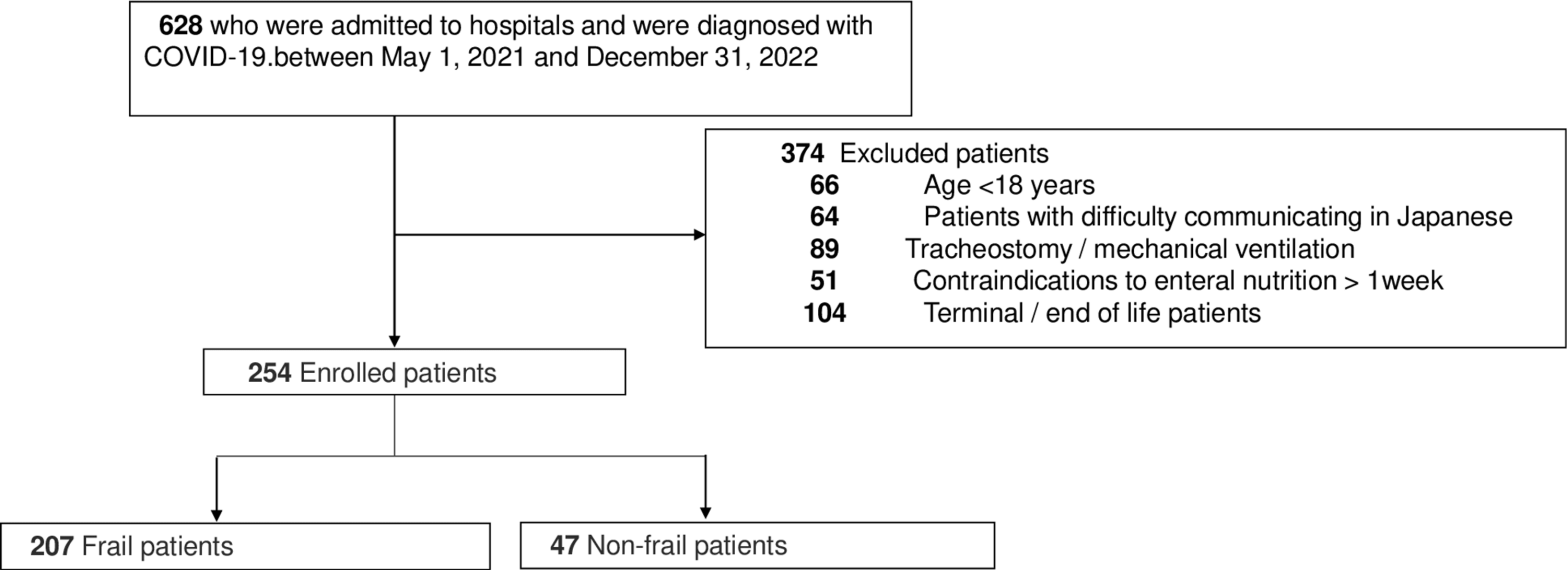
Flow chart of the patient selection process

Herein, 254 patients who met the eligibility criteria but did not meet the exclusion criteria during the study period were assessed and classified as 207 frail and 47 nonfrail patients (Figure 2).

Regarding patient characteristics at the time of admission, significant differences were observed between the frail and nonfrail groups in terms of age (p < 0.001), body mass index (p < 0.001), Charlson Comorbidity Index (p < 0.001), clinical frailty scale (p < 0.001), and Barthel Index at hospital admission (p < 0.001) (Table 2).

**Table 2:** Comparison of items related to baseline characteristics Median (25th-75th percentile) or the number of patients. BMI, body mass index; FOIS, functional oral intake scale

	Total (n = 254)	Frail (n = 207)	Nonfrail (n = 47)	P-value
Age (years)	82 (73-88)	84 (74-88)	76 (66-82)	<0.001
Gender (male), n (%)	136 (54)	111 (54)	25 (53)	1.000
BMI (kg/m^2^)	20 (18-23)	20 (18-23)	23 (20-26)	<0.001
Charlson comorbidity index	2 (1-4)	3 (1-4)	2 (0-3)	<0.001
Covid-19 severity, n (%)				
1	115 (45)	91 (44)	24 (51)	0.508
2	41 (16)	34 (16)	7 (15)	
3	96 (38)	81 (39)	15 (32)	
4	2 (1)	1 (1)	1 (2)	
Quarantine days (day)	11 (10-14)	11 (10-12)	11 (10-16)	0.142
Clinical frailty scale	6 (4-7)	7 (5-7)	2 (1-3)	<0.001
FOIS at hospital admission	4 (1-6)	4 (1-5)	6 (4-7)	<0.001
Barthel index before hospitalization	40 (5-95)	20 (0-70)	100 (95-100)	<0.001

Primary and secondary outcomes

For the primary outcome, the mean SLT time was significantly higher in the frail group than in the nonfrail group (p < 0.001) (Table 3).

**Table 3:** Comparison of primary and secondary outcomes Median (25th–75th percentile) or the number of patients. SLT, speech-language-hearing therapy; FOIS, functional oral intake scale

	Frail (n = 207)	Nonfrail (n = 47)	P-value	Adjusted P-value
Primary outcome				
Mean SLT time (minutes/day)	20 (17-20)	0 (0-20)	<0.001	<0.001
Secondary outcome				
Total SLT time (minutes)	140 (60-240)	0 (0-120)	<0.001	<0.001
Total SLT days (days)	7 (3-12)	0 (0-6)	<0.001	<0.001
Time to first SLT day (day)	2 (1-5)	2 (1-3)	0.240	0.365
FOIS improvement	64 (31)	10 (21)	0.216	0.084
FOIS at hospital discharge	4 (1-5)	7 (5-7)	<0.001	<0.001
Hospital-acquired pneumonia, n (%)	20 (10)	0 (0)	0.030	0.003
Hospital length of stay (day)	27 (16-42)	25 (15-32)	0.135	0.068
Discharge to home, n (%)	41 (20)	27 (57)	<0.001	<0.001
In-hospital mortality, n (%)	30 (15)	4 (9)	0.348	0.753

The secondary outcomes, the total SLT time (p < 0.001), total SLT days (p < 0.001), FOIS score at hospital discharge (p < 0.001), incidence of hospital-acquired pneumonia (p = 0.003), and discharge to home (p < 0.001) was significantly higher in the frail group than in the nonfrail group. No significant differences were observed between the two groups in terms of FOIS improvement or in-hospital mortality.

SLT time and contents from weeks one to four

Table 4 lists the mean SLT time (min/day), total SLT time, SLT days, and SLT content from weeks one to four. The SLT time was significantly longer in the frail group than in the nonfrail group throughout the study period. The frail group performed significantly more indirect and direct swallowing exercises than the nonfrail group throughout the study period. No significant differences were observed between the two groups in the cognitive, speech, or articulation exercises.

**Table 4:** SLT time and contents from weeks one to four Median (25th–75th percentile) or the number of patients. SLT, speech-language-hearing therapy

	Frail (n = 207)	Nonfrail (n = 47)	P-value
1 week			
Mean SLT time (minutes/day)	20 (0-20)	0 (0-20)	<0.001
Total SLT time (minutes)	40 (0-60)	0 (0-20)	<0.001
Total SLT days (days)	2 (0-3)	0 (0-2)	<0.001
Indirect swallowing exercise, n (%)	118 (57)	11 (23)	<0.001
Direct swallowing exercise, n (%)	90 (44)	8 (17)	<0.001
Cognitive exercise, n (%)	9 (4)	3 (6)	0.469
Speech and articulation exercise, n (%)	9 (4)	3 (6)	0.469
Two week			
Mean SLT time (minutes/day)	20 (0-20)	0 (0-20)	<0.001
Total SLT time (minutes)	40 (0-80)	0 (0-40)	<0.001
Total SLT days (days)	2 (0-4)	0 (0-3)	<0.001
Indirect swallowing exercise, n (%)	110 (53)	11 (23)	<0.001
Direct swallowing exercise, n (%)	100 (48)	8 (17)	<0.001
Cognitive exercise, n (%)	13 (6)	7 (15)	0.047
Speech and articulation exercise, n (%)	16 (7)	7 (15)	0.155
Three week			
Mean SLT time (minutes/day)	20 (0-20)	0 (0-20)	0.005
Total SLT time (minutes)	20 (0-80)	0 (0-20)	0.009
Total SLT days (days)	1 (0-4)	0 (0-1)	0.009
Indirect swallowing exercise, n (%)	77 (37)	7 (15)	0.003
Direct swallowing exercise, n (%)	63 (30)	8 (17)	0.073
Cognitive exercise, n (%)	14 (7)	5 (11)	0.361
Speech and articulation exercise, n (%)	12 (6)	4 (8)	0.507
Four week			
Mean SLT time (minutes/day)	0 (0-20)	0 (0-0)	0.035
Total SLT time (minutes)	0 (0-40)	0 (0-0)	0.036
Total SLT days (days)	0 (0-2)	0 (0-0)	0.037
Indirect swallowing exercise, n (%)	48 (23)	5 (11)	0.072
Direct swallowing exercise, n (%)	37 (18)	6 (13)	0.516
Cognitive exercise, n (%)	7 (4)	3 (6)	0.400
Speech and articulation exercise, n (%)	7 (3)	3 (6)	0.399

## Discussion

The causes of swallowing disorders in patients with severe COVID-19 include frail, taste disorders, muscle weakness due to activity restrictions, and cranial nerve abnormalities [20,21]. However, few studies have assessed the content of dysphagia rehabilitation, such as the extent to which the mean SLT time and total SLT days necessary for SLT increase in severe patients. This study investigated the mean SLT time, total SLT days, and SLT exercise in patients with COVID-19.

A study by Katie et al. on daily SLT time, oral intake, and mortality found that approximately 80% of STs reported being unable to provide necessary interventions to patients due to the COVID-19 pandemic. Contributing factors included a lack of manpower, restrictions on the use of personal protection equipment, and the risks linked to aerosol-generating procedures. Similarly, Anna et al. noted that the decrease in the number of SLT clinical cases due to the COVID-19 pandemic was due to various factors, such as time lost due to infection control measures, workplace closures, fewer patients, and staff redeployment to emergency medical fields due to the concerns regarding the risk of infection [22]. This survey indicated that the shortage of ST staff and infection control measures limited the number of interventions, likely increasing the number of daily SLT sessions for more severely frail patients.

Previous studies have shown that frailty onset is closely related to dysphagia [23]. Additionally, dysphagia is a risk factor for nutritional disorders in patients with COVID-19 [24]. Since nutritional disorders result in further muscle loss and decreased oral function and coordination, which have a negative effect on swallowing function [25], a vicious cycle of frail and dysphagia is inevitable [26]. Anna et al. stated that frail may also contribute to dysphagia symptoms in patients with COVID-19 and mentioned the requirement for evaluation and training by SLT [22]. In the present study, the FOIS score at discharge was substantially lower in the frail group than in the nonfrail group. Contrastingly, the difference between the two groups in the improvement of the FOIS was insignificant, which represents an improvement from admission to discharge. This was attributed to the longer daily SLT time in the frail group; that is, the focus of the SLT intervention on frail patients allowed them to achieve the same improvement as nonfrail patients.

The mortality rate of patients with COVID-19 was shown to be higher in frail patients than in nonfrail patients [27]. In the present study, aspiration pneumonia was substantially more common in the frail group; however, no substantial difference in hospital mortality was observed between the two groups. Furthermore, the proportion of swallowing training for frail patients was high. Indirect and direct training included oropharyngeal care and dietary adjustment, respectively. John et al. stated that oral care reduces pneumonia incidence, the number of days with fever, and mortality [28]. While we were unable to examine the course of aspiration pneumonia or nutritional status, in-hospital mortality in frail patients could be reduced to the same level as in nonfrail patients via focused SLT intervention.

This study has several limitations, and the findings should be interpreted with caution. As a multicenter retrospective study, it had a limited survey period and sample size. We recognize the inherent limitations of a retrospective study design, including the potential for selection bias. To mitigate this, we implemented standardized data collection procedures across the participating hospitals. A common protocol was followed for SLT interventions, with predefined assessment criteria based on the Swallowing Clinical Practice Guidelines 2018 and the Expert Consensus on Early Rehabilitation. Additionally, all participating speech-language therapists were trained to adhere to uniform documentation standards, ensuring consistency in data reporting. This study was conducted in two hospitals in Japan, limiting the applicability of our findings to other health systems. Cultural and socioeconomic factors affect the provision of SLT in different ways. In Japan, rehabilitation services are covered by health insurance, which may facilitate longer-term and more intensive SLT interventions compared with health systems with limited rehabilitation coverage. In addition, Japanese hospitals often emphasize multidisciplinary collaboration in the management of dysphagia, which may affect the availability and frequency of SLT. In contrast, health systems in other countries may have different staffing models, reimbursement policies, or rehabilitation priorities, which may lead to variations in SLT practice. Furthermore, CFS was a clinical judgment-based tool and may be subject to inter-rater variability. While other frailty measures such as the Fried criteria could provide an objective assessment, they were not feasible in our study due to the retrospective nature of data collection and the lack of necessary parameters (e.g., grip strength and walking speed) in the medical records. The frail and nonfrail groups were not randomly assigned, limiting the generalizability of the association of SLT in patients with COVID-19 to the general population of COVID-19 patients. While the time required for the SLT was evaluated, the time required for each exercise could not be evaluated, and the mean SLT time could not be fully validated. Additionally, being a retrospective cohort study, proving a causal relationship between frail and nonfrail groups and the time required for SLT was impossible. However, conducting randomized studies based on more evidence remains challenging due to ethical considerations regarding groups that do not receive interventions. Given that conducting a randomized study was not feasible, we opted for a cohort study as the most suitable research design. Thus, increasing the number of cases to verify these results in future studies is necessary.

## Conclusions

The daily SLT time and total SLT days for which SLT was performed were considerably longer in the frail group, with higher rates of direct and indirect swallowing exercises. Further intervention studies are necessary to investigate the causal relationship between COVID-19-related dysphagia and the mean SLT time.
